# Inhibitory Effects of Fosmidomycin Against *Babesia microti in vitro*

**DOI:** 10.3389/fcell.2020.00247

**Published:** 2020-04-28

**Authors:** Sen Wang, Muxiao Li, Xiaoying Luo, Long Yu, Zheng Nie, Qin Liu, Xiaomeng An, Yangsiqi Ao, Qin Liu, Jiaxu Chen, Yu Tian, Junlong Zhao, Lan He

**Affiliations:** ^1^State Key Laboratory of Agricultural Microbiology, College of Veterinary Medicine, Huazhong Agricultural University, Wuhan, China; ^2^Key Laboratory of Preventive Veterinary Medicine in Hubei Province, Wuhan, China; ^3^National Institute of Parasitic Diseases, Chinese Center for Disease Control and Prevention, Shanghai, China; ^4^Key Laboratory of Animal Epidemical Disease and Infectious Zoonoses, Ministry of Agriculture, Huazhong Agricultural University, Wuhan, China

**Keywords:** *Babesia microti*, fosmidomycin, DXR, isoprenoid, babesiosis, methylerythritol 4-phosphate

## Abstract

*Babesia microti*, the main pathogen causing human babesiosis, has been reported to exhibit resistance to the traditional treatment of azithromycin + atovaquone and clindamycin + quinine, suggesting the necessity of developing new drugs. The methylerythritol 4-phosphate (MEP) pathway, a unique pathway in apicomplexan parasites, was shown to play a crucial function in the growth of *Plasmodium falciparum*. In the MEP pathway, 1-deoxy-D-xylulose 5-phosphate reductoisomerase (DXR) is a rate-limiting enzyme and fosmidomycin (FSM) is a reported inhibitor for this enzyme. DXR has been shown as an antimalarial drug target, but no report is available on *B. microti* DXR (BmDXR). Here BmDXR was cloned, sequenced, analyzed by bioinformatics, and evaluated as a potential drug target for inhibiting the growth of *B. micorti in vitro*. Drug assay was performed by adding different concentrations of FSM in *B. microti in vitro* culture. Rescue experiment was done by supplementing 200 μM isopentenyl pyrophosphate (IPP) or 5 μM geranylgeraniol (GG-ol) in the culture medium together with 5 μM FSM or 10 μM diminazene aceturate. The results indicated that FSM can inhibit the growth of *B. microti* in *in vitro* culture with an IC50 of 4.63 ± 0.12 μM, and growth can be restored by both IPP and GG-ol. Additionally, FSM is shown to inhibit the growth of parasites by suppressing the DXR activity, which agreed with the reported results of other apicomplexan parasites. Our results suggest the potential of DXR as a drug target for controlling *B. microti* and that FSM can inhibit the growth of *B. microti in vitro*.

## Introduction

Parasites of the genus *Babesia* are prevalent apicomplexan pathogens transmitted by ticks and infect many mammalian and avian species (Yabsley and Shock, 2013). Human babesiosis is primarily caused by the parasite *Babesia microti*, with most people being infected by ticks and some by blood transfusion (Goethert et al., 2003; Hildebrandt et al., 2007; Young et al., 2012). The infection is characterized by fever and hemolytic anemia and can result in death in severe cases from complications, such as heart failure, respiratory distress, and pulmonary edema (Rosner et al., 1984). Due to the increasing number of people infected with *Babesia*, *B. microti*-related infection has been classified as a nationally notifiable disease since 2011 by the Center for Disease Control (United States) (Herwaldt et al., 2011). Babesiosis is usually treated with atovaquone and azithromycin, but resistance to these drugs has been reported (Krause et al., 2000; Wormser et al., 2010; Simon et al., 2017). Therefore, it is very urgent to develop new anti-*Babesia* drugs.

Apicomplexan parasites contain a vestigial plastid called the apicoplast (McFadden et al., 1996), which plays an important role in the biosynthesis of isoprenoid precursors, fatty acids, and part of the heme (Ralph et al., 2004). However, the apicoplast of *Babesia* is only found in isoprenoid biosynthesis (Brayton et al., 2007; Silva et al., 2016). Apicomplexan parasites utilize the methylerythritol 4-phosphate (MEP) pathway to get isopentenyl pyrophosphate (IPP) and dimethylallyl pyrophosphate (DMAPP) (Imlay and Odom, 2014), which are the basic units of synthetic isoprenoids and essential for parasite growth (Gershenzon and Dudareva, 2007).

Isoprenoids comprise a large family and have an important function in membrane structure, cellular respiration, and cell signaling (Gershenzon and Dudareva, 2007). IPP in living organisms can be synthesized by two pathways [mevalonate (MVA) pathway and MEP pathway] (Odom, 2011). Humans use the MVA pathway to synthesize IPP from acetyl-CoA (Endo, 1992). However, there is no MVA pathway in the genus of *Apicomplexa*, which thus synthesizes IPP by the MEP pathway (Cassera et al., 2004). The MEP pathway was first reported to be present in *Plasmodium falciparum* in 1999 (Jomaa et al., 1999). With the deepening of research, the MEP pathway was found to be crucial for parasites (Cassera et al., 2004). For instance, the deoxyxylose-5-phosphate reductoisomerase (DXR) of *P. falciparum* was shown to contribute to the erythrocyte stage, and inhibiting the DXR activity reduced the growth and the development of the parasites (Odom and Van Voorhis, 2010; Zhang et al., 2011). Additionally, by knocking out the DXR genes of *Toxoplasma gondii*, the parasites were found unable to survive, proving the essentiality of the MEP pathway for their survival (Nair et al., 2011).

The first dedicated step in MEP isoprenoid biosynthesis is accomplished by the bifunctional enzyme DXR (Imlay and Odom, 2014). DXR is competitively inhibited *in vitro* by the antibiotic fosmidomycin (Koppisch et al., 2002; Sangari et al., 2010). Fosmidomycin has been shown to be a clinical prospect for antimalarial drugs due to its inhibition on the recombinant *Plasmodium* DXR to kill *Plasmodium*, and the current clinical trial of malaria treatment with clindamycin is in phase II (Olliaro and Wells, 2009). *Babesia* and *Plasmodium* have many similarities, and they both live in red blood cells (RBCs). In this study, we have found that *B. microti* DXR (BmDXR) has conserved binding sites of fosmidomycin (FSM), and FSM can inhibit the growth of *B. microti in vitro*, suggesting its potential as a new anti-*Babesia* drug.

## Materials and Methods

### Parasites

A *B. microti* strain ATCC PRA-99TM^®^ (Ruebush and Hanson, 1979) was obtained from the National Institute of Parasitic Diseases, Chinese Center for Disease Control and Prevention (Shanghai, China), and maintained in our laboratory (State Key Laboratory of Agricultural Microbiology, College of Veterinary Medicine, Huazhong Agricultural University, China). The parasites were isolated at parasitemia of 30–40% as determined by Giemsa staining of thin blood smears.

### RNA Extraction and cDNA Synthesis

Total RNA was extracted from infected blood by using the TRIZOL reagent (Invitrogen, Shanghai, China) and treated with RNase-free DNase I (TaKaRa, Dalian, China). RNA concentration was measured by NanoDrop 2000 (Thermo, China). The cDNA was prepared from 1 μg of the total RNA using a PrimeScript^TM^ RT reagent kit with gDNA eraser (TaKaRa, Dalian, China).

### Cloning of the BmDXR Gene

Primer pairs of BmDXR were designed based on the sequences of the *B. microti* strain R1: BmDXR-F (5’-ATGACAAATTATTT AAAACTC-3’) and BmDXR-R (5’-TTAACACTTAATTTTTTT TGC-3’). Complete sequences of the BmDXR were amplified by PCR from cDNA separately. The PCR reaction was performed at 95°C for 5 min, followed by 35 cycles of 95°C for 30 s, 47°C for 30 s, 72°C for 1 min 30 s, and finally at 72°C for 10 min. The PCR products were purified and ligated into the cloning vector pEASY-Blunt (Trans, Beijing, China). Three positive colonies of each gene were sent for sequencing analysis by Invitrogen (Shanghai, China).

### Sequence Analysis

The amino acid sequence of BmDXR was aligned with the selected amino acid sequences from other organisms by MAFFT online^1^, then edited by BioEdit v7.25, and phylogenetically analyzed by using the Maximum Likelihood method in MEGA 7 (Kumar et al., 2016). The structure of BmDXR was predicted by SWISS-MODEL^2^ (Guex et al., 2009; Bienert et al., 2017; Waterhouse et al., 2018). The 3D structure of BmDXR was virtually docked with FSM through Molecular Operating Environment (MOE) version 2014.09 (Chemical Computing Group).

#### *B. microti* Short-Term *in vitro* Cultivation

To cultivate *B. microti in vitro*, infected RBCs and healthy mouse RBCs were collected in tubes containing EDTA-2K solution (solution/RBCs = 1:9; 10% EDTA-2K), followed by centrifugation to pellet the cells at 1,000 *g* for 10 min at room temperature), two washes in PSG solution, resuspension of RBCs in the same volume of PSG + G solution, and storage at 4°C until use. The infected RBCs were diluted with healthy RBCs to 3%, followed by cultivation in the presence of HL-1 supplemented with 10 μg/mg AlbuMax I (Gibco Life Technologies), 1% HB101 (Irvine Scientific, Shanghai, China), 200 μM L-glutamine (ATLANTA Biologicals, Shanghai, China), 2% antibiotic/antimycotic 100 × (Corning, Shanghai, China), and 20% fetal bovine serum at 37°C in a microaerophilous stationary phase (5% CO_2_, 2% O_2_, and 93% N_2_) (Abraham et al., 2018).

#### Fosmidomycin Treatment and Rescue Assay

Drug stock solutions of FSM (Sigma-Aldrich, Shanghai, Chain) and diminazene aceturate (DA) (Sigma-Aldrich, Shanghai, Chain) were prepared in sterile water. Geranylgeraniol (Sigma-Aldrich, Shanghai, Chain) stocks were prepared in 100% ethanol. Isopentenyl pyrophosphate triammonium salt solution (Sigma-Aldrich, Shanghai, Chain) was used directly without any additional treatment. For the growth inhibition assay, *B. microti* cultures (20 μl of RBCs plus 100 μl of culture medium) were grown in 96-well flat-bottomed plates, and the susceptibility of *B. microti in vitro* to FSM was evaluated at concentrations up to 500 μM. The results were further confirmed by the IC50 values calculated using the Käber method. All the experiments were repeated three times.

In the rescue experiments, IPP or geranylgeraniol (GG-ol, alcohol of geranylgeranyl diphosphate) was added to the medium containing different drugs. IPP is one of the products in the MEP pathway (He et al., 2018), and GG-ol is the alcohol analog of the downstream isoprenoids (Yeh and DeRisi, 2011; Imlay and Odom, 2014). DA was used as a positive control, and ethanol was used as a negative control. The group of control is only medium. Each drug test was performed in triplicate.

In order to test the parasitemia, three smears were prepared from each well after 72 h of incubation. After air-drying, thin blood smears were fixed with methanol, followed by staining with Giemsa (Sigma-Aldrich, Shanghai, China), and measuring the parasitemia by microscopy. The data were analyzed using GraphPad Prism 7 (San Diego, CA, United States) by two-way analysis of variance (ANOVA), followed by Tukey’s multiple-comparison test. The results are shown as mean ± SD (NS, *P* > 0.05 not significant at 5%; ^∗^*P* < 0.05 significant at 5%; ^∗∗^*P* < 0.01 significant at 1%; and ^∗∗∗^ *P* < 0.001 significant at 0.1%; error bars represent the standard deviations).

## Results

### Cloning and Characterization of *B. microti* DXR

The open reading frame of BmDXR was cloned from *B. microti* PRA99 cDNA by conventional PCR. The results showed that BmDXR is 1,401 bp in length, encoding 466 amino acids with a predicted size of 51.8 kDa. The sequence was submitted to GenBank, with accession number MK673989. BLASTn indicated that BmDXR PRA99 (MK673989) is identical to that of *B. microti* R1 strain (XP_021338225).

### Bioinformatic Analysis

**FIGURE 1:**
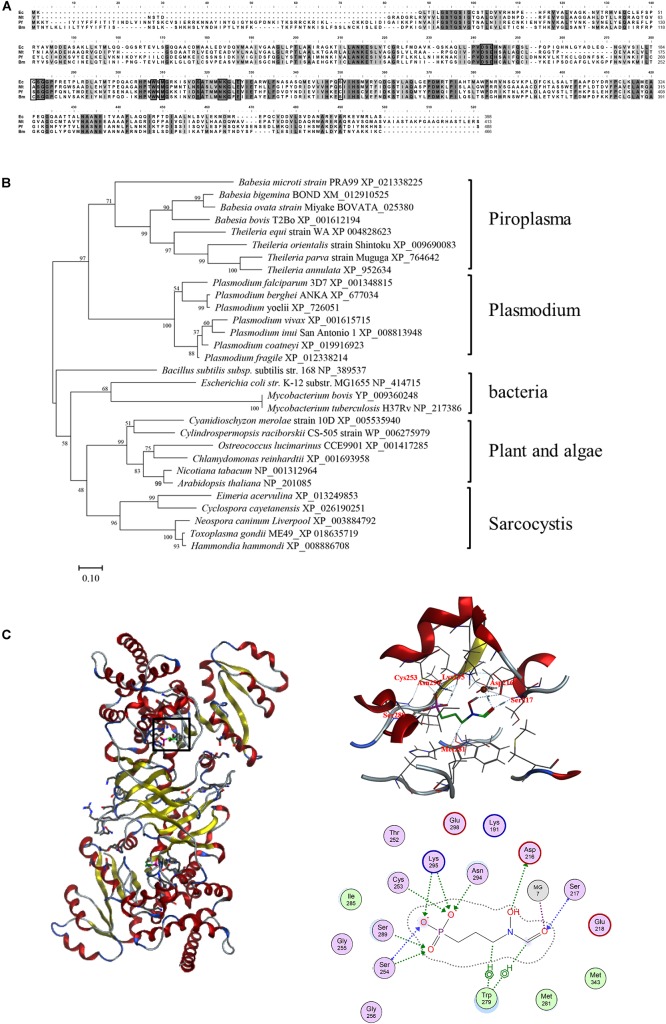
Bioinformatics analysis of the amino acid sequence of DXR. **(A)** Multiple alignment of DXR amino acid sequences. Ec, *Escherichia coli* (NP_414715); *Mt*, *Mycobacterium tuberculosis* (NP_217386); *Pf*, *Plasmodium falciparum* (AAD03739); *Bm*, *Babesia microti* (XP_021338225). Black shading indicates a similarity in four or in more than four species; gray shading indicates a similarity in three species. Black pane indicates the reported fosmidomycin binding site. **(B)** Neighbor-joining phylogenetic tree based on DXR amino acid sequences. The organism names and sequence accession numbers are indicated. **(C)** Prediction of the structure of BmDXR by SWISS-MODEL. The 3D structure of BmDXR is virtually docked with FSM through MOE2014.0901, and FSM can form hydrogen bonds with Ser217, Asp216, Cys253, Met281, Ser289, Asn294, and Lys295 of BmDXR.

The obtained BmDXR sequence was characterized by bioinformatic analysis. SignalP4.1 analysis indicated that BmDXR has a 22-amino-acid signal peptide in N-terminus^3^, and a 48-amino-acid transit peptide right after the signal peptide. The amino acid sequence of BmDXR was aligned with the DXR amino acid sequences of other apicomplexan parasites by MAFFT. The results showed that BmDXR has the highest similarity to the DXR sequence of *P. falciparum* (AAD03739) with a percent identity of 41.71%, and the lowest similarity to that of *Mycobacterium tuberculosis* (NP_217386), with a percent identity of 36.59% (Figure 1A).

DXR amino acid sequences were characterized by phylogenetic analysis with MEGA6, and *B. microti* was shown to fall in the piroplasma clade in the same category of *Plasmodium*. In contrast, bacteria, plant, algae, and sarcocystis are grouped in the same category (Figure 1B). In the piroplasma clade, *B. microti* is significantly different from the other species, including *B. bigemina*, *B. ovata*, *B. bovis*, *T. equi*, *T. orientalis*, *T. parva*, and *T. annulata.*

The 3D structure of BmDXR was predicted by SWISS-MODEL, and BmDXR is shown as a dimeric structure with a metal ion binding site consisting of amino acids D216, E218, and E298. The 3D structure of BmDXR was virtually docked with FSM using MOE2014.0901. The results showed that FSM can form hydrogen bonds with Ser217, Asp216, Cys253, Met281, Ser289, Asn294, and Lys295 of BmDXR (Figure 1C).

### Fosmidomycin Inhibits the Growth of *B. microti in vitro*

**FIGURE 2:**
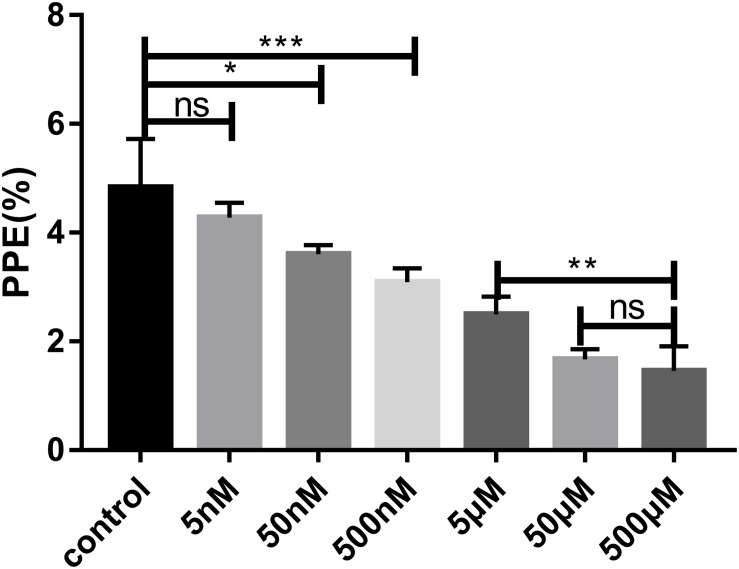
Parasitemia of different drug concentrations after 72 h of treatment. Evaluation of the susceptibility of *B. microti in vitro* to fosmidomycin at concentrations from 5 to 500 μM. Giemsa staining assay at 72 h after the addition of the drug. PPE, pollen productivity estimate; Ns, not significant (*P* > 0.05); *P < 0.05, **P < 0.01, ***P < 0.001.

The effect of FSM on the growth of *B. microti in vitro* was tested by adding different concentrations of FSM into the *in vitro* culture medium at an initial percent parasitized erythrocytes (PPE) of 3%. Parasitemia was counted at 72 h post-treatment by microscopy. The parasitemia of the FSM groups is 4.27 ± 0.28%, 3.60 ± 0.16%, 3.09 ± 0.25%, 2.49 ± 0.33%, 1.67 ± 0.18%, and 1.45 ± 0.45% at the concentration of 5, 50, and 500 nM and 5, 50, and 500 μM, respectively, in contrast to an increase from 3% to 4.83 ± 0.8% for the negative control group (the group without drug) after 72 h of culture. After the 72-h treatment, the parasitemia is significantly lower (*P* < 0.05) in the 50 nM FSM group than in the negative control group, with a significant difference (*P* < 0.01) between 5 and 50 or 500 nm FSM groups, but no difference between the 50- and 500-μM FSM groups (Figure 2). The test results indicated that the drug efficacy is dose dependent, and FSM could not completely inhibit the growth of *B. microti* even at a drug concentration as high as 500 μM (inhibition rate of 70%). Compared to the negative control group, FSM exhibited a potential anti-*B. microti* activity at a low micromolar concentration, with an IC50 of 4.63 ± 0.12 μM.

### IPP and GG-ol Can Rescue *B. microti* Treated by Fosmidomycin

**FIGURE 3:**
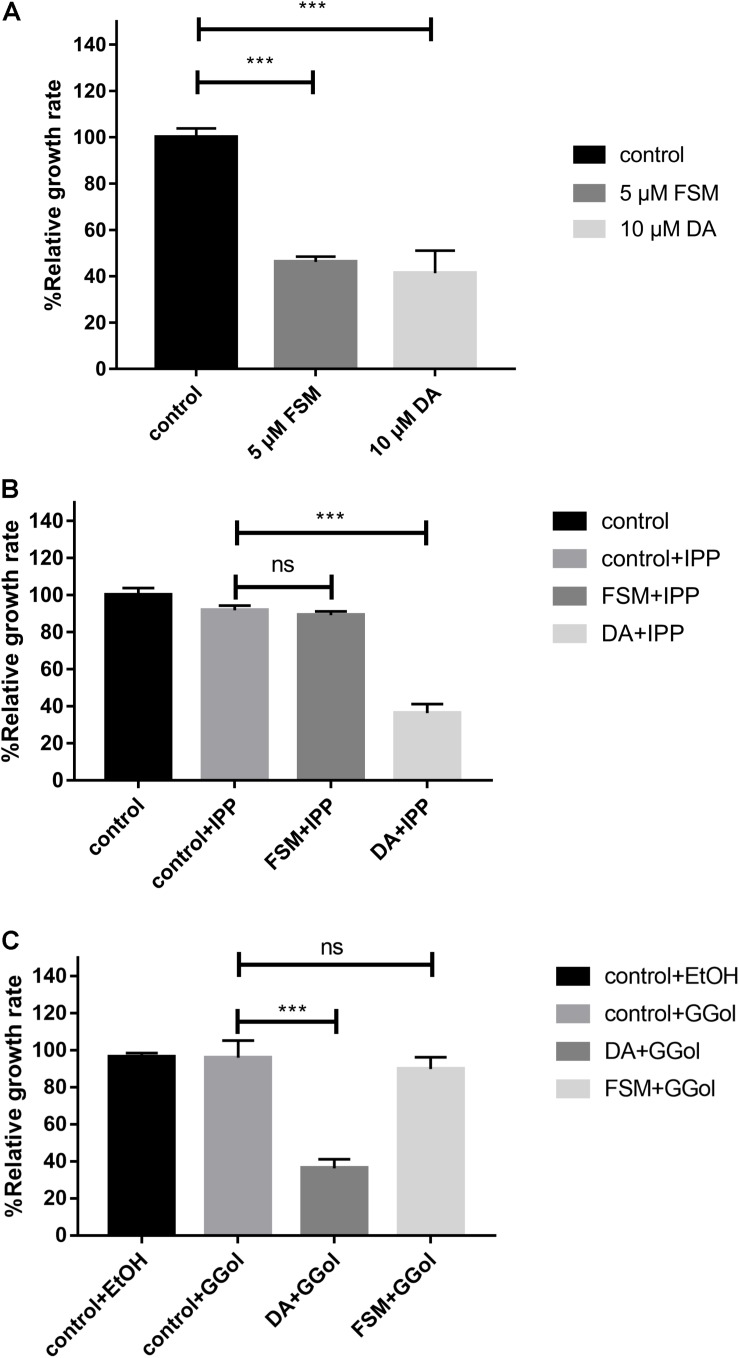
IPP and GGol can rescue *B. microti* treated by fosmidomycin and Giemsa staining assay at 72 h after the addition of the drugs. **(A)** Effect of 5 μM fosmidomycin and 10 μM diminazene aceturate on the growth of *B. microti in vitro*. The group of control is only medium. **(B)** Rescue of *B. microti* by adding isopentenyl pyrophosphate. **(C)** Rescue of *B. microti* by adding geranylgeraniol. ***P < 0.001.

The inhibition of FSM on the growth of *B. mitroti* was investigated through rescue experiments in *B. microti in vitro* cultivation with 200 μM IPP and 5 μM GG-ol added separately into 5 μM FSM and 10 μM DA using the latter as a positive control. The 5 μM FSM and 10 μM DA showed 53.8 and 58.6% inhibition on the growth of the parasites (Figure 3A) in the rescue experiment. The growth in 5 μM FSM could be restored by adding 200 μM IPP or 5 μM GG-ol into culture media as indicated by having no difference (*P* < 0.001) in the relative growth rate among FSM + IPP, GG-ol, and the control (Figures 3B,C). However, the growth in 10 μM DA could not be rescued by adding IPP or GG-ol, as shown by a significant difference (*P* < 0.001) in the relative growth rate among DA + IPP, DA + GG-ol, and the control [ANOVA, F(2, 6) = 259.2, *P* < 0.0001; ANOVA, F(2, 6) = 65.1, *P* < 0.0001] (Figures 3B,C).

## Discussion

**FIGURE 4:**
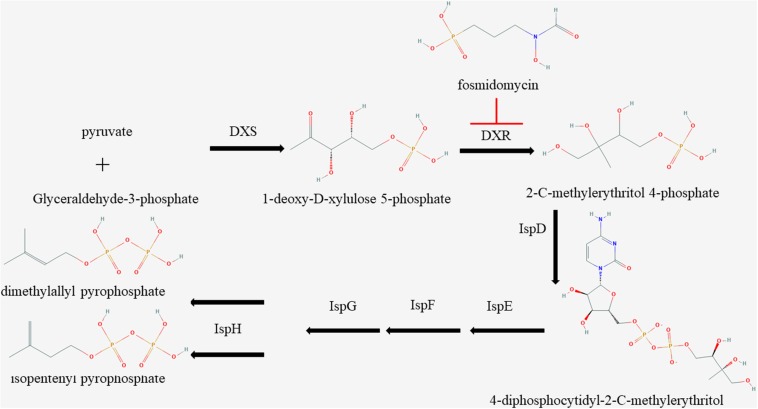
Schematic of isoprenoid metabolism through the non-mevalonate pathway. The methylerythritol 4-phosphate pathway is a unique route to isoprenoid biosynthesis in apicomplexan, and fosmidomycin is a specific inhibitor to 1-deoxy-D-xylulose 5-phosphate reductoisomerase.

The MEP pathway, an essential route in apicomplexan parasites, plays a vital role in the growth of parasites by synthesizing IPP (Imlay and Odom, 2014); however, very few effective inhibitors have been studied. Currently, the MEP inhibitors with lower IC50 for *Plasmodium* are FSM and 1R, 3S MMV008138 (Ghavami et al., 2018). DXR is the second and also a rate-limiting enzyme in the MEP pathway (Imlay and Odom, 2014). The inhibitors of DXR enzymes, such as FSM, suppress the synthesis of IPP in the MEP pathway of multiple organisms *in vitro* (Figure 4; Jomaa et al., 1999). It has been reported that FSM can inhibit *B. divergen*, *B. bovis*, and *B. orientalis in vitro* (Baumeister et al., 2011; Caballero et al., 2012; He et al., 2018). As shown by the reported *P. falciparum* and *M. tuberculosis* crystal structures of inhibitor-free and FSM-bound complete quaternary complexes of DXR (Mac Sweeney et al., 2005; Andaloussi et al., 2011; Umeda et al., 2011), a large cleft was closed between NADPH-binding and catalytic domains upon inhibitor binding, which means that FSM inhibits DXR activity by competing with DOXP. The FSM binding site is conservative, and BmDXR is similar in structure to PfDXR and EcDXR. We speculate that FSM can inhibit the DXR activity in *B. microti* due to its inhibition on the growth of *B. microti* in *in vitro* culture with an FSM IC50 value of 4.63 ± 0.12 μM, which is higher than that of *B. bovis* and *B. bigemina* (3.87 and 2.4 μM, respectively) (Sivakumar et al., 2008). The growth of *B. microti* can be rescued by adding IPP or GG-ol in the culture medium, which agreed with the report that GG-ol can rescue the growth of *B. orientalis* inhibited by FSM (He et al., 2018). These results further suggest that FSM inhibits *B. microti* growth by suppressing the MEP pathway. It is reported that *B. microti*, an obligate parasite of red blood cells (Silva et al., 2016), obtains most of the nutrition materials for parasite survival from host red blood cells, but it cannot obtain IPP from host cells due to the small amount of IPP in RBCs (Wiback and Palsson, 2002). In this case, FSM may inhibit the growth of *B. microti* by suppressing the synthesis of IPP.

FSM can cause the death of *P. falciparum* in the first life cycle (Howe et al., 2013), but we failed to observe the death of *B. microti* after 24 h of treatment at 5 μM FSM. According to the results of Giemsa staining (Supplementary Figure S1), we selected the parasites in the period of merozoites and compared their morphologies. All the merozoites in the control group have an obvious contour and a complete shape, while those treated by FSM have lost their contour and complete shape which, however, can be recovered upon addition of IPP or GG-ol in the medium. This is consistent with the observation in *B. bovis* and *B. bigemina* treated with FSM, with obvious changes in the shape of the parasites (Sivakumar et al., 2008). These results indicate that IPP is important for *Babesia* to keep a normal shape. Meanwhile, the merozoites treated by DA present a pointed shape, which could not be restored to a normal shape after adding IPP or GG-ol. These morphologies indicate a milder efficacy of FSM than DA because DA made the merozoites of *B. microti* point-like, while FSM caused the parasite to lose its normal form. FSM-treated *P. falciparum* was shown to reduce protein prenylation, leading to marked defects in food vacuolar morphology and integrity (Howe et al., 2013). However, no food vacuole has been reported in *B. microti* (Rudzinska et al., 1976), suggesting that the impact of FSM on *B. microti* may be different from its influence mechanism on malaria parasites.

Traditionally, azithromycin + atovaquone was used to treat babesiosis in humans and clindamycin + quinine as a treatment strategy for patients with resistance to atovaquone (Simon et al., 2017). Meanwhile, many patients have adverse reactions to chloroquine (Krause et al., 2000; Rozej-Bielicka et al., 2015). Generally, traditional treatments cannot eliminate *B. microti* parasitemia completely, suggesting the high recurrence potential of *B. microti*. Despite being a safe and effective inhibitor, FSM has some limitations to clinical applications. First of all, it is an unmodified compound which is very costly. Secondly, FSM has a poor pharmacokinetics profile with a plasma half-life of 3.5 h (Na-Bangchang et al., 2007); it will need multiple shots for clinic use. This limitation of FSM can be solved by drug modification. For example, FR9008 is a derivative of FSM, which has a better effect on *P. falciparum* than FSM. Currently, it is necessary to improve the ability of FSM in entering cells and extend its half-life for clinical applications. We believe that the limitations of FSM can be overcome by drug modification. For drug development, modified drugs have better clinical results; for example, dihydroartemisinin has a better effect than artemisinin in treating *Plasmodium* (Li et al., 1983). About combination therapy, clindamycin + FSM can play a better effect in the treatment of *Plasmodium* (Borrmann et al., 2006), but clindamycin has less effect to *B. microti in vitro* (Lawres et al., 2016). Other drugs could be used as combination therapy with FSM if required. Our future work will focus on modifying FSM and the combination therapy of FSM.

## Conclusion

The MEP pathway is a favorable target for drug development. In this study, it is shown that FSM can inhibit the growth of *B. microti in vitro*, which can be rescued by a medium supplemented with IPP or GG-ol. These results indicate that DXR is a potential drug target for designing anti-*Babesia* drugs and that the DXR function and FSM structure contribute to the design of such drugs.

## Data Availability Statement

The datasets generated for this study can be found in the NCBI GenBank under the accession number MK673989.

## Ethics Statement

This study was approved by the Scientific Ethic Committee of Huazhong Agricultural University (permit number: HZAUMO-2017-040). All mice were handled in accordance with the Animal Ethics Procedures and Guidelines of the People’s Republic of China.

## Author Contributions

SW, LH, and JZ designed the study and wrote the draft of the manuscript. ML, XL, LY, ZN, QL, XA, YA, QL, JC, and YT performed the experiments and analyzed the results. All the authors have read and approved the final manuscript.

## Conflict of Interest

The authors declare that the research was conducted in the absence of any commercial or financial relationships that could be construed as a potential conflict of interest.

## Footnotes

1 https://mafft.cbrc.jp/alignment/software/

2 https://swissmodel.expasy.org/

3 http://www.cbs.dtu.dk/services/SignalP/

## Supplementary Material

FIGURE S1 Morphology of merozoites cultured *in vitro* for 72 h. 5 μM FSM and 10 μM DA caused changes in merozoite morphology, and 200 μM IPP or 5 μM GG-ol can restore merozoite morphology treated by FSM.

## Abbreviations

DXR: 1-deoxy-D-xylulose 5-phosphate reductoisomerase
FSM: fosmidomycin
DA: diminazene Aceturate
IPP: isopentenyl pyrophosphate
GG-ol: geranylgeraniol
ORF: open reading frame
MEP: 2-C-methylerythritol 4-phosphate
IC50: half-maximum inhibition concentration
PPE: percent parasitized erythrocytes.
