# Supplementary figures and images for: Inhibitory Effects of Fosmidomycin Against Babesia microti in vitro
Source: Front Cell Dev Biol. 2020 Apr 28;8:247. doi: 10.3389/fcell.2020.00247 (PMC7198706; doi:10.3389/fcell.2020.00247)

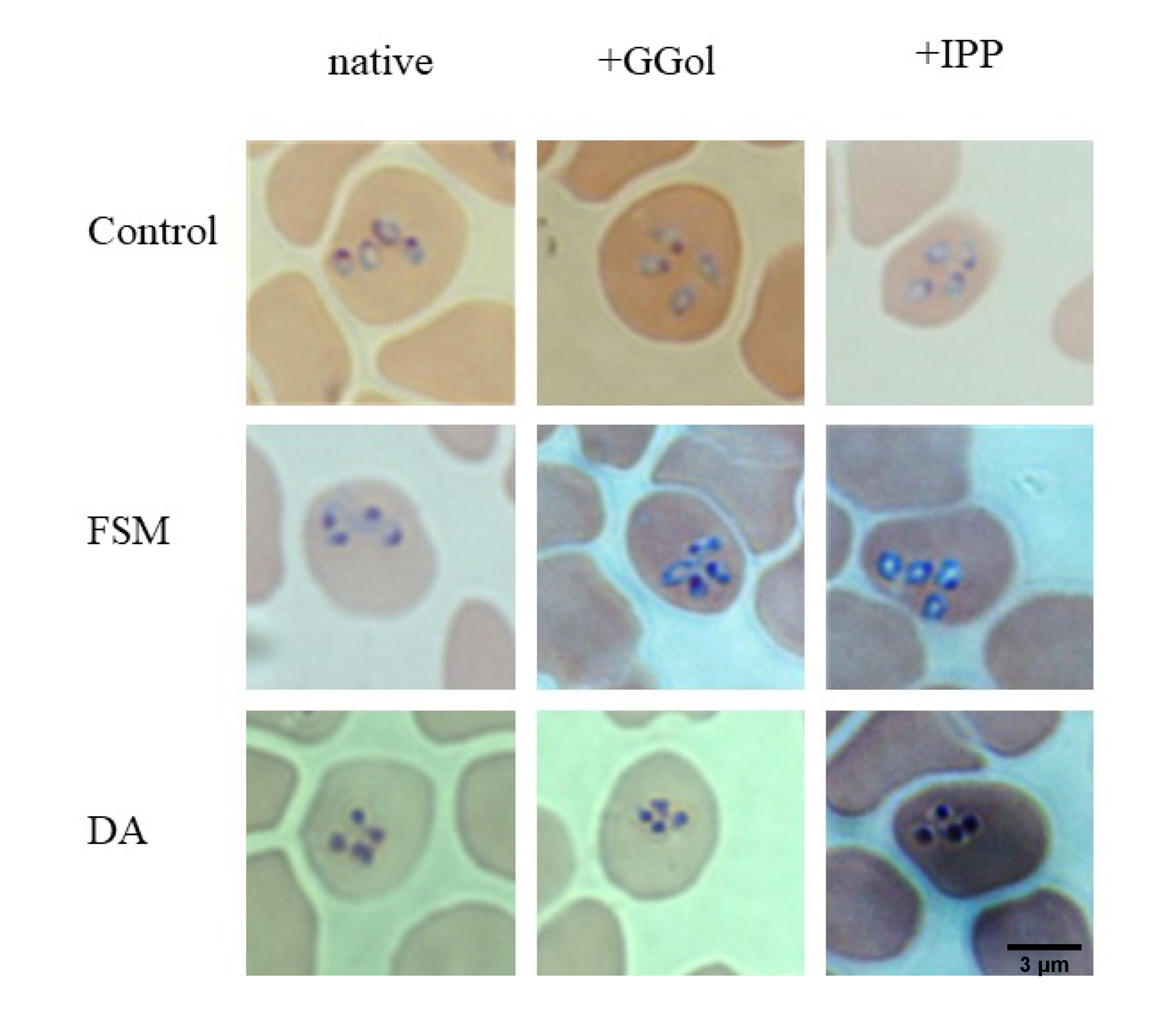

Supplement: FIGURE S1 — Morphology of merozoites cultured in vitro for 72 h. 5 μM FSM and 10 μM DA caused changes in merozoite morphology, and 200 μM IPP or 5 μM GG-ol can restore merozoite morphology treated by FSM. [file Image_1.TIF]
